# The role of managers in addressing employees with musculoskeletal pain: a mixed methods study

**DOI:** 10.1007/s00420-017-1284-1

**Published:** 2017-12-21

**Authors:** Anne Konring Larsen, Signe Falkenstrøm, Marie Birk Jørgensen, Morten Hulvej Rod

**Affiliations:** 1 National Research Centre for the Working Environment, Lersø parkallé 105, Copenhagen, Denmark; 2 National Institute of Public Health, University of Southern Denmark, Copenhagen, Denmark; 3 National Research Center for Disadvantaged Children and Youth, Metropolitan Universitiy College, Copenhagen, Denmark

**Keywords:** Leader, Workplace, Support, Communication, Supervisor

## Abstract

**Purpose:**

This study investigates management awareness of employee musculoskeletal pain and conditions that shape managers’ handling of employees with pain.

**Methods:**

We used a mixed methods design including data from a questionnaire survey and focus group sessions. All employees and managers from seven nursing homes were invited to participate in the questionnaire survey and 327 employees (81%) and 31 managers (82%) responded. Employees were asked about their worst pain intensity the past month and managers were asked to estimate the percentage of their employees who had experienced pain. Thirty-eight managers (93%) participated in focus group sessions addressing the culture for handling pain at the workplace. A multiple case study approach allowed for an integrated interpretation of the empirical findings.

**Results:**

Results indicate limited manager awareness of employee pain. We identified four conditions that shape managers’ handling of employees with pain: (1) Employee handling of—and communication about—pain, (2) the collegial culture for handling pain, (3) managers’ perception of their role towards employees with pain and (4) procedures and informal approaches for handling employees with pain. Across these conditions various degrees of openness characterized the nursing homes.

**Conclusions:**

The degree of openness towards communicating about—and handling pain—in the organization (individual, collegial and managerial levels) influences how managers handle employees with pain. Awareness about employee health is a prerequisite for management to initiate relevant action towards supporting employees. Future workplace initiatives are likely to benefit from addressing openness in the organization to increase awareness and support employees with pain.

## Introduction

Musculoskeletal pain (pain) constitutes a considerable problem, particularly among nursing aides with physically demanding work tasks. Work tasks such as patient handling can substantially increase the risk that pain will have consequences, such as work disability and impaired quality of life (McDonald et al. 2011; Mortensen 2008; Leclerc et al. 2006). Therefore, the interplay between work demands and pain perception can play an important role for nursing aides’ ability to keep their job.

For some employees, pain results in sickness absence or even early retirement pension while other employees attend work while in pain. A Danish study found that more than 70% of the employees in the workforce had been present at work despite pain or sickness during a year (Hansen and Andersen 2008). Attending work with pain is anticipated to be particularly prevalent within care and nursing jobs (Dew et al. 2005) and impact the quality of work conducted (Kronborg et al. 2009). Still a recent study found that management did not consider pain a problem among their employees in nursing homes (Rasmussen et al. 2014).

Various factors have been suggested to influence whether an employee decides to attend work or call in sick when experiencing pain. For example, the culture for handling pain at the workplace, health promotion initiatives at the workplace, and how the individual copes with pain (Dellve et al. 2007; Barnes et al. 2008; de Vries et al. 2011). Thus, factors at both the individual, interpersonal and organizational levels may influence employee action when experiencing pain.

Furthermore, management behavior can influence how an employee handles pain at work (Dellve et al. 2007; Sterud et al. 2014; Wynne-Jones et al. 2011). Particularly within job groups with high physical work demands and low degree of influence among employees, management is suggested to play a central role for whether an employee can continue working when experiencing pain, for example, by providing possibilities for trustful communication about employee health and possibilities for adjustments of work tasks according to employee health (Linton et al. 2016; Johansson and Lundberg 2004).

Even though managers seem to play a key role in supporting employees with pain, a little is known about management awareness of pain among their employees. Furthermore, only few studies have investigated management perspectives and how they address employee pain. Previous studies found that managers report lack of competences to handle employees with pain (Linton et al. 2016; Cunningham et al. 2008; Shaw et al. 2006), but more research is needed in this area. Therefore, the overall aim of this study is to shed light on conditions that shape managers’ handling of employees with pain including an investigation of whether management has knowledge about employee pain, the culture for employee handling of pain and managers attitudes and behavior towards employees with pain in Denmark.

## Methods

### Research design

We used a mixed methods design and included both qualitative (focus groups) and quantitative (questionnaire) data sources. The study was conducted using baseline data of an organizational workplace intervention described elsewhere (Larsen et al. 2015). Both the survey and the focus group sessions were embedded in a formative evaluation on each workplace as part of the initial phase of the intervention.

To illuminate the overall aim of the study, we investigated the following research questions: (1) Are managers aware of the scope of pain among their employees? (2) How do managers perceive employees’ handling of pain? (3) How are managers’ attitudes and behavior towards supporting employees with pain? The quantitative data provided knowledge about managers’ awareness of employee pain and the qualitative data allowed for a more in depth investigation of the managers’ awareness and perceptions of employee pain as well as their attitudes and behavior towards supporting employees with pain.

### Sample and recruitment

Seven nursing homes participated in the study. All nursing homes were located within two municipalities in the Eastern region of Denmark and were both public and private nursing homes of various sizes and with different management structures. Participants received information about the study through information meetings and e-mails. Middle managers working at the level just above the individual nursing aides and nursing assistants from the seven nursing homes took part in the focus group sessions. Participants were primarily women; however, some of the nursing homes had one male middle manager. The majority of the middle managers were nurses but also middle managers from technical groups and kitchen participated. The nurse managers had between 3 and 3 ½ years of specific nursing training and in some cases no formal management training. Employees were primarily nursing aides or nursing assistants with 1 or 2 years of nursing aid training, respectively. The job tasks for these groups are predominantly similar; however, there are tasks only nurses’ assistants are certified to handle, for example, medicine. All employees and managers at the nursing homes were invited to participate in the survey.

### Data collection

The qualitative data consists of seven focus groups with 38 out of the 41 middle managers. One focus group was held at each nursing home with participation from three to eight middle managers. All focus group sessions lasted approximately 1 hour and were conducted at the nursing homes during working hours. One or two researchers from the research group conducted the sessions, all were female and previously experienced in interviewing. Semi-structured interview guides were used and participants were encouraged to talk and interact with each other and to explore and shed light on individual and shared perspectives (Krueger 2000).

The quantitative data consists of data from questionnaires, collected using mobile phone text messages. The question for employees was: “During the previous 4 weeks, on a scale from 0 to 10, what was the highest intensity of pain in your muscles and joints? (0 = no pain, 10 = worst imaginable pain)” and the question for the managers was: “In percent, how many of your employees do you think have experienced pain in muscles and joints within the last 4 weeks (answer in percent from 0 to 100)?” The data were collected between October 2013 and December 2014 and both data sources were collected over a period of 2 months at each nursing home. The questionnaires were administered to all participants whether they were at work (for example, on sick leave) unless they specifically asked us not to.

### Analysis

We used a multiple case study approach as described by Yin et al. where each nursing home represents a case with data from both quantitative and qualitative sources, so that seven cases were analyzed to capture the key elements related to the overall aim of the study and each research question (Yin 2009).

The methodological combination of qualitative and quantitative data allowed us to shed light on middle managers’ awareness and handling of employees with pain from different perspectives. To answer the first research question, the quantitative data from employees and managers were analyzed descriptively and compared. In the quantitative analysis of management knowledge about employee pain, we asked the managers to estimate how many of their employees they thought had pain in muscles and joints at all (i.e., pain > 0) within the last month. To describe the concordance between employee pain and managers’ estimates, we used the cut point of employee pain ≥ 4. This cut point was chosen based on the previous literature which suggests that pain above this threshold predicts increased sickness absence and bothersomeness at work (Andersen et al. 2012). In Table 2, we present the agreement between employee pain and managers’ estimates. The agreement is calculated by dividing managers’ estimates with the percentage of employees with pain ≥ 4 (%) at each nursing home, these numbers are presented in Table 2.

The focus group sessions were recorded and transcribed. Two persons read through each of the focus group interviews with special attention to statements related to each of the research questions. Based on the relevant statements in each interview, a summary of the main points related to each question was made by each person. The summaries were compared to assess whether there was a general agreement and if there were discrepancies. Any discrepancies were discussed and explored further if necessary. Both persons read through all cases and identified and noted themes that had emerged in each case.

**Fig. 1:**
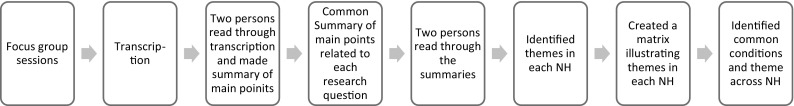
Illustrating the process of analyzing the qualitative data from the focus group interviews, and identification of main points related to each question, to identifying themes, to creating a matrix and finally the common themes across nursing homes. (*NH* nursing home)

**Fig. 2:**
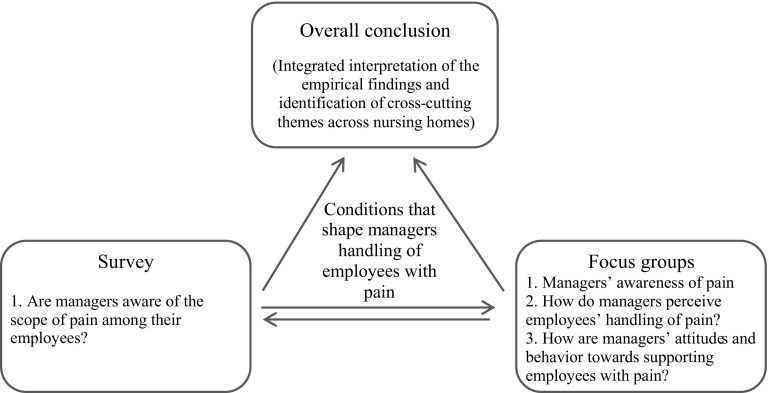
Illustrating the overall aim of the study, and the combination of the qualitative and quantitative methods, the research questions and how the identification of cross-cutting themes and integrated interpretation led to the overall conclusion

Based on this material we identified general themes across the nursing homes, i.e., conditions that influenced middle managers’ handling of employees with pain and we constructed a matrix in which each case had a column and every condition had a row. This allowed for focus on each nursing home but also for an overview of each condition across nursing homes. See Fig. 1 for an illustration of the steps in the analysis. To fully utilize the potentials of the mixed methods design, we finalized the analysis by an integrated interpretation of the empirical findings to identify common cross-cutting themes across nursing homes (Tashakkori and Teddlie 2003; Bryman 2007). Figure 2 illustrates the overall aim of the study in the center and the data sources with the research questions at the empirical level (lowest) and the integration and interpretation of these findings at the theoretical level (in the top). The entire process was conducted by two researchers and continuously discussed until consensus.

## Results

### Nursing homes descriptive characteristics

A total of 523 persons (482 employees and 41 managers) were employed at the nursing homes at the time of data collection, ranging from 50 to 105 employees and between three and eight managers. The employees were divided into teams working together in departments at the nursing homes. The six smallest nursing homes had a management three-level structure with one top manager and a number of middle managers and the employees. At the largest nursing home, there was one additional management level and thus there was a top manager, group leaders that had more administrative tasks and middle managers with the direct contact to employees. Each middle manager was responsible for one or two teams and each team comprised between 1 and 41 employees, with a mean of 16 employees.

Out of the 523 employees and managers working in the participating nursing homes, 85% were signed up to receive a questionnaire (406 employees and 38 managers) and 79 were for different reasons not signed up, for example, if they had not disclosed their phone number or if they did not want to participate. Out of these 444 persons, 327 employees (81%) and 31 managers (82%) answered the questionnaire. The mean age among employee respondents was 47 years, mean seniority was 7 years and 89 percent were female. Among non-respondents, the mean age was 49 years, mean seniority was 6 years and 87 percent were female. Table 1 illustrates the mean age, sex and seniority among respondents (managers and employees) within each nursing home. The mean age among employees was between 46 and 50 years, mean seniority was between 5 and 8 years and between 78 and 96 percent were female. Among managers mean age was between 45 and 59 years, mean seniority was between 4 and 15 years and between 67 and 100 percent were female.

**Table 1:** Illustrates mean age, seniority and sex for respondents (employees and managers). We do not have information about seniority in NH 5

Nursing home	Employees (*n*)	Employee age (mean years)	Employee seniority (mean years)	Employee sex (% female)	Managers (*n*)	Managers’ age (mean years)	Managers’ seniority (mean years)	Managers’ sex (% female)
1	40	47	5	80	4	49	6	100
2	47	46	7	88	4	51	6	100
3	37	50	7	92	4	56	15	50
4	47	47	8	96	6	45	10	83
5	43	49		92	3	59		100
6	66	47	8	91	3	52	11	66
7	47	47	5	78	7	49	4	86
Total	327	47	7	89	31	50	8	88

In the following, results from each research question will be presented. After all citations an indication of the nursing home will follow, for example, (NH 2) indicates a citation from nursing home 2.

### Research question (1) Are managers aware of the scope of pain among their employees?

As presented in Table 2, the data indicate differences between the nursing homes, both in regard to the proportion of employees reporting pain as well as the managers’ estimates. Between 35 and 70% of the employees in the nursing homes report that they have experienced pain ≥ 4 on a scale from 0 to 10 within the last 4 weeks (average pain level between 3,0 and 5,0). The managers’ estimates of the percentage of their employees that have experienced pain within the last 4 weeks range between 14 and 52%. As illustrated in Table 2, some nursing homes seem to have a higher agreement between the employee report of pain and the managers’ estimations. The percentage of employees that reported experiencing pain at all within the past month was approximately 80%.

**Table 2:** Employee worst pain, the percentage of employees with pain at or above 4 and managers estimates of percentage of employees with pain, the agreement between managers’ estimates of percentage of employees with pain and percentage of employees that report pain

Nursing home	Employees (*n*)	Managers (*n*)	Employee worst pain (SD) 0–10 scale)	Employees with pain ≥ 4 (%)	Managers’ estimates of employee pain (%) (SD)	Agreement between managers’ estimates and employee pain (%)
1	40	4	4.3 (2.7)	60	14 (13)	23
2	47	4	3.5 (2.9)	45	28 (10)	62
3	37	4	3.0 (3.1)	35	35 (44)	100
4	47	6	3.8 (3.1)	53	18 (19)	34
5	43	3	5.0 (3.0)	70	52 (13)	74
6	66	3	3.9 (2.9)	56	35 (22)	63
7	47	7	4.9 (3.0)	64	30 (22)	47
Total	327	31	4.1	55	30	58

We further explored this question with the qualitative data. These data indicate that the middle managers are quite ambiguous in their statements of the extent of problems regarding pain among the employees. At most of the nursing homes, the middle managers say that they think only a small proportion of the employees experience pain. As one middle manager stated:

*“I think it is rarely we experience employees with pain” (NH 3)*

Moreover, some middle managers argue that the workplaces have the necessary ergonomic equipment, and therefore, the work should not be physically demanding and cause pain among the employees.

At the same time, however, most middle managers express that they do have some employees who experience problems with pain and are absent from work due to pain.

*“Of course physical pain also has an impact on sickness absence; once in a while there are employees who call in sick due to pain in the back or in the shoulder…”(NH 4)*

At another nursing home, the middle managers state that they do not think they know enough about the extent of problems with pain among their employees. And that they would like to know more about the problems among their employees.

*“I do not know enough about the problem of pain among my employees” (NH5)*

This statement is given by the middle managers at a nursing home with a relatively high level of agreement between managers’ estimates and employee reported pain.

### Research question (2) How do managers perceive employee handling of pain?

#### Individual handling of—and communication about—pain

The middle managers describe that they observe considerable individual differences in how the employees handle pain, for example, some employees stay at home when they experience pain while others come to work.

*“some (employees) might think it will pass tomorrow. And some think: ’Ohh no, I need to be sicklisted, I have to go to the doctor…’ It is very dependent on the person how you handle it” (NH 4)*

According to the middle managers, the willingness to communicate about these issues also varies between employees, some employees are open to sharing their problems about pain issues and work environment challenges, while other employees do not inform the middle managers about their problems at all, or at least not until it is too late. The middle managers mention three potential explanations why employees do not share challenges regarding pain; some employees deny that they experience pain, some are afraid of losing their job while others expect the middle managers to discover the problem themselves and act.

In one nursing home, the middle managers express that the employees do not give the managers a chance to help them because they do not share their problem until it is too late.

*[In response to the question whether employees inform the managers when they experience pain] “Yes, I think so. They do. Then you know that the sick leave is coming soon”. “Yes, but it is actually not until that time!” (NH 6)*

Furthermore, several middle managers express that the employees do not take enough responsibility towards communicating their problems, instead some employees place the responsibility of acknowledging the problems and acting on the managers or colleagues.

*“…recently (I) had an employee who came walking like this [she demonstrates and sighs] and did not say anything. ‘Are you okay?’ – ‘no, no, it is the back and it is completely off’, where they kind of wait: ‘you are my manager, so you have to legitimize that I’m ill. You have to tell me that it is okay.’ No, stop it I think. Take that responsibility yourself”. (NH 3)*

Some middle managers point out that it is likely that some employees do not inform the managers about pain issues early because they fear getting fired. Therefore, some employees deny that they have any problems even if the middle managers confront them.

*“It seemed like they got worried about whether or not they would be discharged or… I think it seemed like they got a bit worried and did not want to talk about it… what I found most notable was they were not interested in talking about what can we do about it? It was almost like, no I definitely do not (experience pain)!” (NH 6)*

Thus, according to the middle managers, employees do not on a regular basis share their problems regarding pain.

#### The collegial culture for handling pain

The middle managers describe that colleagues can have both a positive and a negative effect on how the employees prevent and handle pain. The middle managers in some of the nursing homes say that the employees are good at helping each other with the heavy tasks:

*“I also think they are good at swapping tasks. Figuring out who they can take instead to avoid those bad strains” (NH 4)*

Some middle managers experience that colleagues sometimes take upon them the responsibility of other employees, when they notice a colleague in pain, and encourage them to go home.

*“I guess we are also characterized by the fact that we are a female-dominated workplace so the solicitude sometimes gets out of hand: ‘My Good, you look bad, what are you doing here?’” (NH 3)*

At the same time, according to the middle managers, seeing colleagues go home because of pain or attend work only for a few hours can also cause frustration among employees because no extra resources is provided to fulfill the work tasks and so the remaining employees have to do extra tasks.

*“The employees often feel’Then she is here for two hours, but it is the rest of us that have to take over and do her work. So taking someone [employees] back for a few hours can also result in disputes within the staff ‘And then she just went home and we have to continue slogging’” (NH 2)*

At one nursing home, the middle managers have experienced that the employees are reluctant to help each other and ask for help when handling overweight patients. The middle managers have observed that there is a norm that you are weak if you cannot handle your patients alone.

*“Scarily I now experience, that there is some kind of culture out there…’shut up…you wimp…do you really need help with that - I will take it…!” (NH 2)*

This statement is followed by a discussion in the focus group about how to change this culture and strengthen the individual responsibility towards colleagues and the common task of providing good care for the habitants. Most middle managers in the other nursing homes express that their employees generally are willing to help each other. However, some of the middle managers point out that the communication between the employees sometimes is rough and could be improved. For example, one manager says,

“I don’t believe it! Is that really a way to talk to each other …you really should not stand and shout at your colleagues, you have to be conscious about how you communicate with others, I think” (NH 7)

Summing up it seems that the middle managers have quite different perceptions of the cultures among the employees for handling colleagues with pain both in regard to the communication between coworkers and the action and support towards colleagues with pain.

### How are managers’ attitudes and behavior towards supporting employees with pain?

#### Managers’ perception of their role towards employees with pain

The middle managers express diverse attitudes both in regard to how they expect their employees to act when they experience pain and how they themselves act towards employees with pain. Some middle managers believe that it is their responsibility to evaluate employee health and act, while others expect the employees to take the responsibility and do not consider it a matter for the workplace. Finally, some middle managers practice being a role model for the employees. These attitudes are reflected both in the communication with and the handling of employees with pain.

While all middle managers describe spending a lot of time talking to employees every day, the picture is different when it comes to communicating about pain. Some middle managers wish for more organized structures and procedures for communicating about work environment and pain with their employees to ensure that they receive the relevant information about these specific issues from the employees.

*“I could imagine that we could easily be more systematic in our approach to the employees, so when they get they…experience pain somewhere, then you might as a manager reach the employee earlier and say, what can we do – also to avoid that the employee suddenly calls in sick. And…exactly the thing with talking… what can I do – well as a manager. And there I think that you can get a system made and that way around prevent that we get sick leave due to pain” (NH 2)*

Other middle managers are reluctant to talk about pain with the employees because they believe that “what you focus on, you will get more of” so if you talk about pain, the employees will start thinking more about whether they experience pain, and that will cause more pain-related problems among the employees.

*“…then you sit and focus on “did I have pain? And you almost forgot that you were in pain, but “yeah I had”, and “how much pain did I have?” and things like that”(NH 3)*

Other middle managers experience that talking to the employees and helping them in solving their problems can be quite demanding and sometimes simply too much. Therefore, they emphasize the necessity to sometimes take care of themselves and not let the employees get too close.

*”And then I also think that we have some young employees who are very vulnerable. Also mentally right. So you have to be real good at pushing them away”. (NH 7)*.

This difficult balance between wanting to help the employees and the necessity to take care of yourself is supported by the other managers in this focus group and another manager continues:

*“Yes you really have to think about what is this about and be strong enough to push them away. And then nevertheless on a day where everything is going fast then you still get involved in the problem and trying to solve it…” (NH 7)*

Some of the other middle managers concur with this attitude and some are even very clear that they think it is the employees who have the responsibility for taking care of themselves. They acknowledge that some employees experience pain, but do not believe that the workplace should solve these problems.

*“We acknowledge if they have pain, but it is not a problem for the work place to solve”… “Well if an employee comes to work we assume they are grown up people and capable of evaluating [whether they are well enough]”. (NH 3)*

At another nursing home, the middle managers say that they, to some extent, prefer that employees come to work despite having pain, so that they (the managers) can evaluate whether the employees are ill enough to go home.

*“It is kind of us, who need to interpret the situation and feel where something is going on.”… “Then your manager can go in and say: ‘no, you look bad today, go home’” (NH 4)*

Several of the middle managers describe that they see themselves as role models for the employees, trying to act as they want the employees to act:

*“it also has a lot to do with the fact that we have to be those role models and that is just a part of the job…” (NH 1)*

One example as to how the middle managers see themselves as role models is according to some middle managers that they personally handle pain by taking painkillers and carry on with their work tasks and they encourage the employees to practice the same.

Summing up, we have identified three overall positions regarding middle manager roles: (1) the manager that takes the responsibility of evaluating employee pain and acting upon it, (2) the manager that places the responsibility on the employee and (3) the “role model” manager that acts as she wishes the employees to do.

#### Procedures and informal approaches for handling employees with pain

Most middle managers express that they have no systematic procedures for either communicating about—or handling—employees with pain. As one middle manager explains:

*“.. it is somewhat implicit in the everyday life and quite random whether we talk about it and act on it (employee problems regarding pain)” (NH 5)*

Furthermore, several managers express that they lack tools to support employees with pain.

*“…I don’t know how to handle employee s with pain. I have no idea. I have no idea how to handle my own sometimes, right.”…I cannot see what I can do for the employees. But if I have to do something, then I need tools for doing so” (NH 7)*

At the same time, most middle managers say that they do act and try to support employees when they learn about employee problems regarding pain. The managers describe some informal structures, considerations and approaches for handling employees with pain. These vary between nursing homes and depending on the individual situation, but include according to the managers, for example, adjustment of employee work tasks and work time, encourage employees to do physical training, use a positive mindset, and provide time off to consult a professional.

*“Yes, I do believe that I do so…and if they kind of tell you that they need to go to some kind of treatment or do something else, then we try to make sure that they can take the time off”(NH 4)*

In regards to adjusting work task one manager explains:

*” then I say that they should take some of the more light tasks and then go a little easier through it today” (NH 6)*

According to the middle managers, they usually cannot call in additional personnel, regardless of whether some employees call in sick or are present at work, but in pain. Therefore, when adjusting work tasks in favor of one employee, consequently, another employee has to work harder. Still according to the middle managers, it is a good solution if the employee can contribute a little bit rather than nothing at all. The middle managers emphasize the importance of constructively communicating this to the colleagues, because otherwise adjusting work tasks could be a source of frustration and conflicts between colleagues, as mentioned earlier in the section on collegial culture.

*“So that they (the colleagues) could understand it, we kind of had to gather the team, and get something on writing; what is it that Amalie can and cannot do, so that it was visible to them! Because otherwise it became a lot like; ‘why is she just sitting there or why do she need to sit again or…’” (NH 1)*

At some of the nursing homes, the middle managers take the responsibility of guiding the employees towards professionals that can help them in their specific situation such as doctors, physiotherapists.

*“But I also think that we as managers have to be good at saying: remember to go to the physiotherapist” …”And encourage them, now you simply must go to the doctor. It is no use you just go and ignore it. Get something done about it…” (NH 4)*

At one nursing home, the middle managers say that they explain to the employees how a positive attitude instead of negative, can actually result in a better day for themselves, their colleagues and the residents. Saying, for example,

*“You decide yourselves how the day is going to be”. Well if we chose to go to the residents with “Oh my god, how annoying”, then that is going to control the day and your state of mind and the result of that. If we chose to say, well okay – as a starting point – then we have an approach that says, we will make the best out of it. And smile a little about it…” (NH 6)*

Hence, there are no standardized procedures for handling employees with pain, still the middle managers point out many different informal approaches to help and support employees.

## Discussion

This discussion contains an integrated interpretation of the empirical findings of this study and a discussion of the findings of this study in relation to the existing literature.

The aim of the study was to shed light on middle managers’ attitudes and behavior towards employees with pain and conditions that shape middle managers’ handling of employees with pain. The main findings of the study were that managers have limited awareness of employee pain. However, we found considerable variations between nursing homes both in regard to percentage of employees with pain and managers’ awareness. Furthermore, the results points at some central conditions, at the individual employee level, the collegial and the managerial level, that influence how managers handle employees with pain:


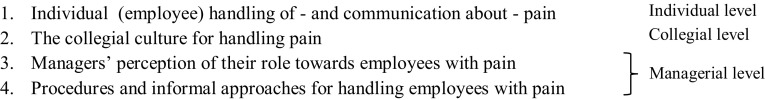


**Fig. 3:**
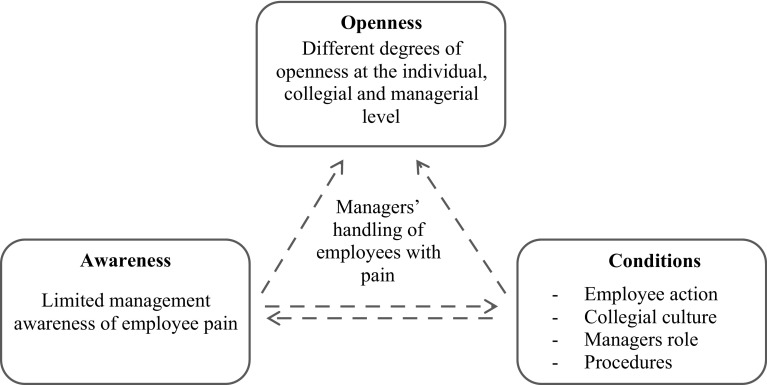
Illustrates our integrated interpretation of the quantitative and qualitative empirical findings (two lower boxes) and at the theoretical level (the top of the triangle) the appearance of the cross-cutting theme of openness in the different levels in the organization

Our integrated interpretation of the results across the identified conditions revealed a cross-cutting theme of openness in the communication, culture and procedures to manage pain as an important characteristic that varies between nursing homes (see Fig. 3). Meaning that at each nursing home the culture is characterized by different degrees of openness towards communicating with and handling employees with pain.

The degree of openness towards communicating about and handling pain differ between organizations, but it also varies within organizations meaning that the level of openness in the nursing homes varies between the management level, colleagues and the individual level.

Openness is previously found to be connected with trust in organizations and associated with employee performance, retention and quality of care in the health care sector (Okello and Gilson 2015). Furthermore, specific supervisor openness towards employees has shown to be connected with employee safety (Tucker and Turner 2015). And a study by van Scheppingen et al. found that implementation of increased dialogue and communication between employees and between employees and managers increased the openness about health at these levels in the organization (van Scheppingen et al. 2014). Still how openness at the different levels of the organization impacts the management of pain in the organization remains to be studied.

Our results generally indicate limited management awareness of pain among employees. This is in line with the findings of Rasmussen et al. that management does not consider pain a problem among their employees (Rasmussen et al. 2014). Limited management awareness of employee pain is discussed previously in a few studies and one suggested reason for this is insufficient communication between management and employees about work environment challenges and health (Pransky et al. 1999; Wynne-Jones et al. 2011). Our study suggests that lacking communication regarding pain may be caused either by the employees’ assumptions regarding the consequences or the managers’ willingness to talk about it. For example, the employees may fear possible consequences of sharing their problems with the management. This barrier for communication is also identified in a previous study by Tveito et al. who found that some employees did not disclose their problems regarding pain to their employers because they felt they would be unwilling to accommodate their needs and they were worried that their employment would be terminated (Tveito et al. 2010). The managers may have limited willingness to talk about pain because they believe talking about it will reinforce the scope of the problem or because they believe that it is an individual responsibility to solve problems regarding pain. Their willingness to talking about pain is associated with the degree of openness in the culture or organization around the topic of pain.

The awareness among the managers varies between nursing homes and there seems to be a higher agreement between the employee report of pain and the managers’ estimations in some of the nursing homes. We could not identify specific common characteristics across these nursing homes that could explain the higher awareness of employee pain among the managers. These nursing homes range from having a very open to less open cultures among managers towards communicating about and handling pain. At some nursing homes with high manager awareness of pain, the managers express that they have an open culture and that they want to communicate about pain and express a wish to know more about employee pain and support their employees. At another nursing home with high manager awareness of pain, the managers describe a less open culture where the managers hesitate to talk about pain with their employees and say that it is principally the responsibility of the employee to handle their pain. The nursing home with the highest agreement between employee pain and managers estimates (NH 3) also differs from the rest of the nursing homes in regard to pain level. Here, the pain level is considerably lower than at the rest of the nursing homes, only 35% of the employees report pain at or above 4. Interestingly, the managers at this nursing home express a very limited degree of openness towards communicating about pain and handling employees with pain. Thus, in this case, we cannot identify an explanation for the high manager awareness of employee pain.

Previous studies have found that management play a crucial role in supporting employees with pain, for example, in how to use the organizational policies and possibilities at the workplace (Wynne-Jones and Main 2011; Wynne-Jones et al. 2011; Elfering et al. 2002; Dellve et al. 2007). There are different possible explanations why we did not find a direct association between high management willingness to communicate about pain and lower pain levels in this case study. One explanation can be that only the organization with high willingness to communicate about work environment and pain, and support employees with pain can accommodate and maintain employees with pain problems at the workplace thus explaining the high prevalence in these organizations. Another possibility is that low management involvement in employee work environment and pain issues somehow facilitates less pain among the employees, for example, by individual empowerment and action. However, that is in contrast to previous studies that found that supervisor support was associated with reduced chronic pain and increased staying at work with pain(de Vries et al. 2012; Sterud and Johannessen 2014). Therefore, further research on the topic will be necessary to understand the consequences of organizational openness for addressing employee pain.

Apart from manager and employee willingness and assumptions regarding talking about pain, other factors may play a role for the management of pain at the workplace. For example, the manager’s competences for pain management are likely to be important. Some managers say that they do not feel they have the competences to support and handle their employees with pain. This is in line with the findings of previous studies where supervisors report lack of competences in dealing with employees with pain problems (Shaw et al. 2013, 2006; Cunningham et al. 2008). This has also been described from the employee perspective where Wynne-Jones et al. found that some employees do not disclose their problems to management because they do not have confidence in the managers’ abilities to help them (Wynne-Jones et al. 2011).Thus, the degree of openness at the employee level might be influenced by management competences.

Previous studies have found that employee communication about their pain problems to management and colleagues has a positive impact on employee management of pain, pain-related sickness absence and health care use (Tveito et al. 2010; Linton et al. 2016). Some managers in this study also express a wish for talking in a more structured manner with employees about work environment and pain. This may indicate that the building of formal structures to discuss pain problems may be beneficial in pain prevention.

Also, in regard to the procedures for handling employees with pain, the middle managers say that they do not have any structured procedures. Nevertheless, they describe a number of informal structures, considerations and approaches such as adjusting the work tasks giving the employee time off to see a professional. Flexibility at work and the possibility to adjust the work tasks according to employee needs is previously found to positively affect both work attendance and employee management of pain at work (Tveito et al. 2010; Dellve et al. 2007). This could suggest advances of organizational openness towards handling employees with pain. According to the Danish Work Environment Act, the employer is responsible for safe and healthy work conditions for their employees. Companies must compile a workplace evaluation to uncover problems in the work environment and develop a plan for action regarding any existing problems at the workplace in general. All participating nursing homes have completed workplace evaluations and thus considering the scope of the problem, managers’ experience of not having a plan for handling employees with pain is thought-provoking.

To sum up, this study contributes with information about management awareness of employee pain. Furthermore, this study adds to the limited amount of the literature on the middle management perspective of employee pain by pointing at specific conditions that influence middle managers’ handling of employees with pain. These conditions involve both the individual employee, the relationships between colleagues, and formal and informal organizational procedures.

### Strengths and weaknesses

Strength of the study is the use of a mixed methods approach allowing for an overall picture of the prevalence and awareness of pain and a more in depth understanding of the existing cultures at the workplaces and middle managers’ considerations about their communication and handling of employees with pain. The strength of using focus group sessions compared to individual interviews is the dynamic that arises in the group during discussions facilitating that different perspectives and arguments are discussed. At the same time there is a risk that some perspectives will be suppressed, viewpoints that might be easier to talk about in one-on-one interviews. When using only interviews with middle managers this study provides an illustration of the middle manager perspective. In light of the findings that the managers’ awareness of employee pain seems to be limited, one could argue that the reliability of their statements regarding employee prevention and handling of pain might also be debatable and uncovering the employee perspective could be an interesting study for future research.

Whether the employee report of pain ≥ 4 within the last month is directly comparable to the managers’ estimates of the percentage of employees experiencing pain within the last month can be discussed. We asked the management how many of the employees they thought had experienced pain at all (i.e., pain > 0) within the last month. The percentage of employees that reported experiencing pain at all within the past month was approximately 80% (compared to 55% reporting pain ≥ 4). We chose the cut point of pain ≥ 4 to capture the employees with pain at a level that is likely to affect their everyday life, based on the literature that suggests that a pain level at or above 3–5 on a 10-point likert scale, predict sickness absence and bothersomeness at work (Andersen et al. 2012).

Comparing the employees’ report of pain and managers’ estimates introduces a risk of systematic bias. A systematic over or underreporting in one of the groups could indicate a greater discrepancy between the groups than is actually there or diminishing a difference that is truly larger. However, the findings do indicate considerable discrepancies between employee reports and management estimates. Physical job demands could influence the extent of pain among employees. We do not have knowledge about differences in the physical job demands at the nursing homes. This should be taken into account when comparing the scope of pain across nursing homes.

Finally, the geographical location of the nursing homes might limit the external validity of the results. In Denmark, there is a relatively low unemployment rate and a focus on inclusion of employees on the labor market. Danish legislation supports retention of employees at the workplace (fx the workplace receives salary refund during employee sickness absence and is obligated to hold sickness absence meetings with the employee to reduce absence and maintain a connection with the workplace). Furthermore, the nursing homes are situated within two municipalities and this clustering of the respondents within the same areas might limit the diversity of both the quantitative and qualitative data. Still the cases do represent various types of nursing homes with regard to, for example, size, management structures and team structures.

## Conclusion

This study found limited management awareness of employee pain; however, there were considerable variations between nursing homes. Conditions at the individual level, the collegial level and the managerial level influence how middle managers handle employees with pain. From integrated interpretation of quantitative and qualitative data, we identified a cross-cutting theme of openness in the communication, culture and procedures to manage pain that seems to be important for the middle managers’ handling of employees with pain. However, we could not identify a connection between the workplace openness the level of employee pain or managers’ awareness of employee pain. Nevertheless, awareness about existing problems at the workplace is a prerequisite for management to initiate relevant action towards supporting employees with pain. Thus, based on these findings implications for future studies to strengthen managers’ possibility for supporting employees with pain are ensuring management awareness of the scope of the problem of pain among employees and focusing on management as well as employee knowledge about handling pain. Furthermore, tools and structures for improving communication about pain and implementation of organizational procedures might improve awareness and suitable, tailored handling of employees with pain.
